# Endothelial Dysfunction in Cystic Fibrosis: Role of Oxidative Stress

**DOI:** 10.1155/2019/1629638

**Published:** 2019-06-19

**Authors:** Matthew A. Tucker, Brandon M. Fox, Nichole Seigler, Paula Rodriguez-Miguelez, Jacob Looney, Jeffrey Thomas, Kathleen T. McKie, Caralee Forseen, Gareth W. Davison, Ryan A. Harris

**Affiliations:** ^1^Georgia Prevention Institute, Department of Population Health Sciences, Augusta University, Augusta, GA, USA; ^2^Medical Scientist Training Program, University of Alabama at Birmingham School of Medicine, Birmingham, AL, USA; ^3^Pediatric Pulmonology, Augusta University, Augusta, GA, USA; ^4^Pulmonary and Critical Care Medicine, Augusta University, Augusta, GA, USA; ^5^Sport and Exercise Science Research Institute, Ulster University, Jordanstown, UK

## Abstract

Oxidative stress and vascular endothelial dysfunction are established characteristics of cystic fibrosis (CF). Oxidative stress may contribute to vascular dysfunction via inhibition of nitric oxide (NO) bioavailability. *Purpose*. To determine if ingestion of a single antioxidant cocktail (AOC) improves vascular endothelial function in patients with CF. *Methods*. In 18 patients with CF (age 8-39 y), brachial artery flow-mediated dilation (FMD) was assessed using a Doppler ultrasound prior to and two hours following either an AOC (*n* = 18; 1,000 mg vitamin C, 600 IU vitamin E, and 600 mg *α*-lipoic acid) or a placebo (*n* = 9). In a subgroup of patients (*n* = 9), changes in serum concentrations of *α*-tocopherol and lipid hydroperoxide (LOOH) were assessed following AOC and placebo. *Results*. A significant (*p* = 0.032) increase in FMD was observed following AOC (Δ1.9 ± 3.3%), compared to no change following placebo (Δ − 0.8 ± 1.9%). Moreover, compared with placebo, AOC prevented the decrease in *α*-tocopherol (Δ0.48 ± 2.91 vs. −1.98 ± 2.32 *μ*M, *p* = 0.024) and tended to decrease LOOH (Δ − 0.2 ± 0.1 vs. 0.1 ± 0.1 *μ*M, *p* = 0.063). *Conclusions*. These data demonstrate that ingestion of an antioxidant cocktail can improve vascular endothelial function and improve oxidative stress in patients with CF, providing evidence that oxidative stress is a key contributor to vascular endothelial dysfunction in CF.

## 1. Introduction

Cystic fibrosis (CF) is the most prevalent autosomal recessive genetic disease in North America. While the shortened life expectancy accompanying the disease can most often be attributed to pulmonary infection [1], patients with CF also suffer from a variety of systemic complications including dysfunction of the gastrointestinal, immune, endocrine, and musculoskeletal systems [2, 3].

The flow-mediated dilation (FMD) technique is a widely used, noninvasive bioassay of conduit vessel endothelial function [4–6] and nitric oxide (NO) bioavailability [7]. Our group has recently provided evidence of both microvascular and conduit artery endothelial dysfunctions in patients with CF [8, 9]; however, the mechanisms that contribute to vascular endothelial dysfunction in this population have yet to be elucidated.

Considerable evidence indicates that systemic oxidative stress is a feature of CF [10–16] and may contribute to the reduction in NO bioavailability and subsequent endothelial dysfunction [17]. In CF, this imbalance between free radical production and neutralization of radicals by antioxidants arises due to the combined effects of persistently elevated immune activation [16, 18] and both dietary deficiency and malabsorption of exogenous antioxidants [11, 16, 18]. Administration of oral antioxidants has been demonstrated to temporarily reduce oxidative stress and improve vascular function in other populations [19–22]; however, the role of oxidative stress in vascular dysfunction in patients with CF is unknown. Therefore, this study sought to test the hypothesis that a single dose of an antioxidant cocktail would reduce oxidative stress and improve vascular endothelial function, whereas no change would be observed following a placebo condition.

## 2. Materials and Methods

### 2.1. Participants

**Figure 1:**
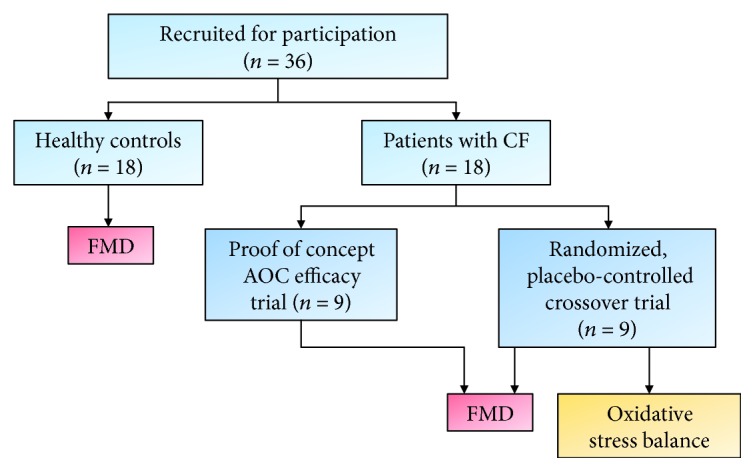
Schematic illustrating the recruitment/enrollment process and overall experimental design. Flow-mediated dilation (FMD) was assessed in healthy controls (*n* = 18) and in patients with CF (*n* = 18) following an antioxidant cocktail (AOC). In a subgroup of patients with CF (*n* = 9), measures of oxidative stress balance were assessed following ingestion of the AOC and a placebo condition.

Figure 1 illustrates the recruitment and testing process for participants in this study. Based on the efficacy of the antioxidant cocktail (AOC) in other clinical populations [20, 22], a proof of concept efficacy trial of the AOC was conducted in 9 patients during one visit. Following this initial study, 9 additional patients with CF were recruited to take part in a double blind, randomized, placebo-controlled, crossover trial where patients received the AOC (CF-AOC) and placebo (CF-PLC) in randomized order on separate experimental visits. Of our patient population, 50% were homozygous F508del, 22% were F508del/G551D, 22% were heterozygous with one copy of F508del, and 11% were heterozygous without F508del. Only the four patients with gating mutations were on modulator therapy (ivacaftor) and had been taking it for at least 3 months prior to testing. To further examine the impact of the AOC on oxidative stress, circulating markers of oxidative stress balance were determined prior to and 2 hours following AOC or placebo treatment. 18 demographically matched (age, sex, height, weight, and BMI) healthy controls were recruited to provide a reference standard of vascular function and to determine the efficacy of the treatment response in patients with CF. The control group did not undergo any treatment nor were any of the pre- to posttreatment biomarkers evaluated.

All patients were enrolled if they had a clinical diagnosis of CF based on positive sweat tests and genotype analysis. Participants were excluded if they (1) had a forced expiratory volume in one second (FEV_1_) < 50% of predicted, (2) had a resting oxygen saturation (SpO_2_) < 85%, (3) self-reported to be a smoker, (4) were diagnosed with pulmonary hypertension, (5) were pregnant or nursing at the time of the investigation, (6) had a clinical diagnosis of cardiovascular disease, hypertension, or CF-related diabetes, or (7) were prescribed any vasoactive medications (i.e., nitrates, beta blockers, and ACE inhibitors). All participants and parents of children provided written and verbal consent/assent prior to participation. All study protocols were approved by the Institutional Review Board at Augusta University. This study was registered to the clinicaltrials.gov website (#NCT01772758).

### 2.2. Experimental Design

All participants reported to the Laboratory of Integrated Vascular and Exercise Physiology (LIVEP) at the Georgia Prevention Institute for a preliminary visit that consisted of the informed consent process, body composition assessments, and a baseline pulmonary function test (PFT). For each of the experimental visits, participants reported to the LIVEP in the morning following an overnight fast and having abstained from moderate to vigorous physical activity for 24 hours prior to investigation. Patients were instructed to adhere to the timing of their daily pulmonary therapy and come to the lab following their morning airway clearance and inhaled medicines. Upon arrival, baseline assessments of PFT and FMD were performed, and a venous blood sample was obtained. Patients were then given either an oral AOC (CF-AOX; 1,000 mg vitamin C, 600 IU vitamin E, and 600 mg *α*-lipoic acid) or a visually similar cocktail of placebo pills (CF-PLC; sucrose or galactose). Following ingestion of treatment, patients rested quietly for two hours, and a posttreatment FMD was performed.

### 2.3. Participant Characteristics and Clinical Laboratory Values

Height and weight were determined using a stadiometer and standard platform scale (CN20, DETECTO©, Webb City, MO) and used for calculations of body mass index (BMI). Body fat percentage, fat mass, and fat-free mass were determined using dual energy X-ray absorptiometry (QDR-4500W; Hologic, Waltham, MA), and resting systolic and diastolic blood pressures were evaluated using established protocols [23]. Resting oxygen saturation was obtained using an Onyx II fingertip sensor (Nonin Medical, Plymouth, MN). Fasting concentrations of total cholesterol (TC), high-density lipoproteins (HDL), low-density lipoproteins (LDL), triglycerides (TG), and glucose were obtained using a Cholestech LDX point of care analyzer (Alere Inc., Scarborough, ME). Hemoglobin and hematocrit were determined using a HemoPoint H2 analyzer (Stanbio Laboratory, Boerne, TX). Concentrations of high-sensitivity C-reactive protein (hsCRP) were obtained from standard core laboratory techniques (Laboratory Corporation of America Holdings, Burlington, NC).

### 2.4. Biomarkers of Oxidative Stress and Lipid Soluble Antioxidants

Markers of oxidative stress balance were determined prior to and following the administration of the AOC and PLC. Plasma concentrations of 8-isoprostane (Cayman Chemical, Ann Arbor, MI) and nitrotyrosine (Cell Biolabs Inc., San Diego, CA) were determined via a colorimetric assay following the manufacturer's instructions. Total serum hydroperoxide (LOOH) concentrations were determined by the ferrous oxidation-xylenol orange (FOX1) assay [24] using a protocol previously described by our group [25].

Serum *α*-tocopherol, *γ*-tocopherol, retinol, and lycopene were determined using high-performance liquid chromatography (HPLC) as previously described in a protocol by our group [25]. Data were analyzed by Empower analytical software (Waters, Ireland).

### 2.5. Pulmonary Function Testing (PFT)

An assessment of pulmonary function was performed using the EasyOne Pro® LAB system (ndd Medical Technologies, Andover, MA) to determine forced vital capacity (FVC), FEV_1_ (L), FEV_1_ (% predicted), FEV_1_/FVC, and forced expiratory flow at 25-75% (FEF_25-75_) in all participants according to the American Thoracic Society standards [26]. Briefly, following the American Thoracic Society recommendations [27], a minimum of three reproducible trials were completed by each participant, and the best of three acceptable forced expiratory maneuvers was used for analysis. The European Respiratory Society Global Lung Function Initiative spirometric reference standards were used to determine the percentage of predicted data set [28].

### 2.6. Flow-Mediated Dilation and Shear Rate

Brachial artery FMD was determined using the Doppler ultrasound (Logiq 7, GE Medical Systems, Milwaukee, WI) performed in accordance with published guidelines [29] and methodology previously described by our group [8, 30]. Briefly, simultaneous B-mode and blood velocity profiles of the brachial artery were evaluated by ultrasound imaging using a 12 MHz linear transducer. After acquisition of baseline values, a forearm occlusion cuff placed immediately distal to the medial epicondyle was rapidly inflated to 250 mmHg for 5 min (E-20 rapid cuff inflator, Hokanson) to induce arterial occlusion and then deflated to induce reactive hyperemia of the brachial artery. R-wave gating (AccuSync 72, AccuSync Medical Research, Milford, CT) was used to capture end-diastolic arterial diameters for automated offline analysis of brachial artery vasodilation (Medical Imaging Applications, Coralville, IA). The greatest 5 s diameter average after cuff release was used as the peak response. FMD was expressed as the percent increase in peak diameter from baseline diameter and also relative to shear rate (FMD/shear).

The cumulative shear rate (area under the curve (AUC (s^−1^))) and FMD/shear were determined as previously described by our group [8, 30]. Absolute change in diameter, peak diameter, and time to peak dilation were calculated and reported according to published guidelines and recommendations [31] to provide a comprehensive assessment of vascular endothelial function.

### 2.7. Statistical Analyses

All analyses were performed using SPSS version 24 (IBM Corporation, Somers, NY). Descriptive statistics were generated, and range as well as normality checks were performed. Independent *t*-tests were performed to identify differences in demographics, clinical laboratory markers, and pulmonary function parameters between patients with CF and healthy controls. Comparisons of baseline (pretreatment) parameters of the FMD test between CF-AOC and CF-PLC groups were performed using independent *t*-tests. A two-way (group by time) ANOVA was used to test for pre- to posttreatment differences in parameters of the FMD test and markers of oxidative stress between AOC and PLC. Covariates related to disease severity (FEV_1_ (% predicted) and HbA1c as an index of glycemic control) were included in the regression model where appropriate. Effect sizes (partial eta squared (*η*_P_^2^)) are reported for the interaction terms of the ANOVA, where values of 0.01, 0.06, and 0.14 correspond to small, medium, and large effects, respectively [32]. Values are presented as the mean ± SD unless otherwise noted. An alpha < 0.05 was considered statistically significant for all analyses.

## 3. Results

### 3.1. Participant Characteristics, Clinical Laboratory Values, and Pulmonary Function

Baseline characteristics, clinical laboratory values, and indices of pulmonary function for patients with CF and healthy controls are presented in Table 1. There were no differences in demographic or anthropometric characteristics between patients and controls; however, patients exhibited significantly lower (*p* < 0.05) TC, and HDL, and significantly higher (*p* = 0.003) hsCRP compared to controls. There were no differences in FVC between groups; however, patients had significantly lower absolute FEV_1_, FEV_1_ (% predicted), FEV_1_/FVC, and FEF_25-75_ versus controls (all *p* < 0.05). In addition, while resting SpO_2_ was at a normal level in patients (98%), it was significantly lower compared with controls (*p* = 0.005).

### 3.2. Flow-Mediated Dilation

**Figure 2:**
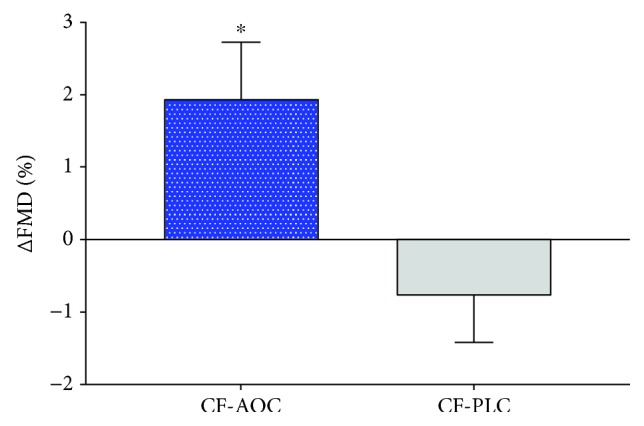
Changes in flow-mediated dilation (FMD) in patients with CF following either the AOC treatment (CF-AOC; *n* = 18) or the placebo (CF-PLC; *n* = 9). ^∗^Significantly greater than CF-PLC (*p* = 0.032). Values are presented as the mean ± SEM.

Figure 2 illustrates a significant improvement (*p* = 0.032, *η*_P_^2^ = 0.170) in FMD following the AOC, whereas no change was observed following the placebo. Additional parameters of the FMD test are presented in Table 2. There was a significant increase (*p* = 0.004) in FMD normalized for shear rate (FMD/shear) and decrease in time to peak dilation (TTP; *p* = 0.011) in CF-AOC and CF-PLC, but changes were not different between groups (*p* = 0.137, *η*_P_^2^ = 0.086 and *p* = 0.288, *η*_P_^2^ = 0.045, respectively). While the change in absolute diameter was significantly greater (*p* = 0.023, *η*_P_^2^ = 0.189) in CF-AOC versus CF-PLC, changes in baseline diameter (*p* = 0.622, *η*_P_^2^ = 0.010), peak diameter (*p* = 0.115, *η*_P_^2^ = 0.096), and shear rate (*p* = 0.820, *η*_P_^2^ = 0.002) were not different between groups.

In addition to the CF patient data, FMD data from demographically matched, healthy participants are also presented in Table 2 as a control reference of normal vascular endothelial function. While pretreatment FMD (%) was not significantly different in CF-AOC or CF-PLC versus controls (*p* = 0.101 and *p* = 0.590, respectively), pretreatment FMD/shear was significantly lower (*p* = 0.010) in CF-AOC versus controls. This deficit, however, was improved following the AOC treatment, leading to restoration in both posttreatment FMD and FMD/shear compared to controls (*p* = 0.660).

There were no differences between CF-AOC or CF-PLC and controls in pretreatment baseline diameter (*p* = 0.463 and *p* = 0.077, respectively), peak diameter (*p* = 0.688 and *p* = 0.126), or absolute change in diameter (*p* = 0.434 and *p* = 0.753); however, TTP was lower in controls versus both CF-AOC (*p* = 0.008) and CF-PLC (*p* = 0.024).

### 3.3. Biomarkers of Oxidative Stress and Lipid Soluble Antioxidants

**Figure 3:**
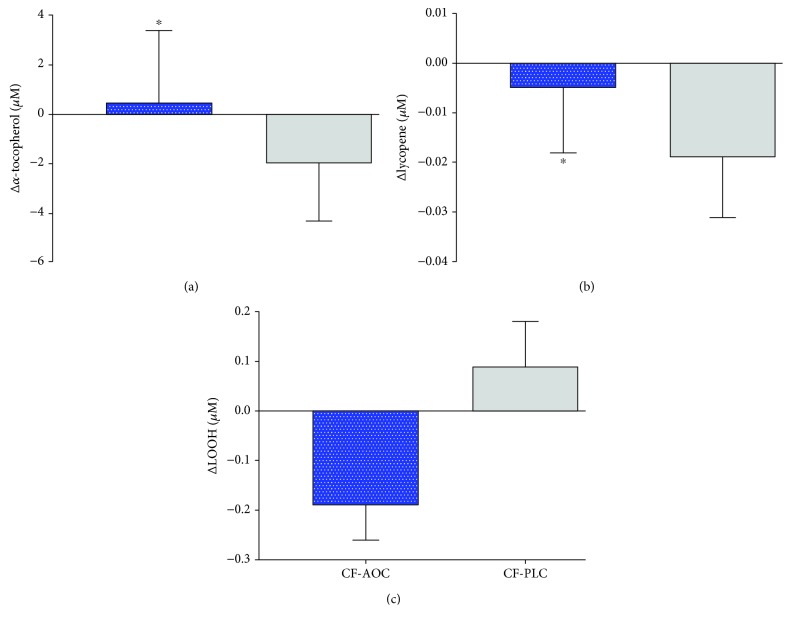
Changes in plasma levels of antioxidants (a, b) and lipid hydroperoxide (LOOH, (c)) in patients with CF in the AOC treatment and placebo (PLC) condition (*n* = 9). ^∗^Significant difference between treatments when controlling for HbA1c as an index of disease severity (*p* < 0.05).

Baseline (pretreatment) levels of *α*-tocopherol (21.7 ± 14.4 vs. 21.3 ± 16.5 *μ*M, *p* = 0.788), lycopene (0.06 ± 0.06 vs. 0.05 ± 0.09 *μ*M, *p* = 0.610), and LOOH (0.74 ± 0.11 vs. 0.87 ± 0.22 *μ*M, *p* = 0.164) were not different between PLC and AOC.  Figure 3 illustrates the change in oxidative stress balance following the AOC or PLC. Specifically, reductions in *α*-tocopherol (*p* = 0.024, *η*_P_^2^ = 0.54) and lycopene (*p* = 0.014, *η*_P_^2^ = 0.60) were significantly attenuated following AOC compared with PLC while controlling for HbA1c. While not significant, LOOH tended to decrease (*p* = 0.063, *η*_P_^2^ = 0.33) following the AOC versus PLC. Additional systemic markers of oxidative stress and lipid soluble antioxidants are presented in Table 3. AOC treatment changes in 8-isoprostane (*p* = 0.815, *η*_P_^2^ = 0.01), nitrotyrosine (*p* = 0.820, *η*_P_^2^ = 0.01), *γ*-tocopherol (*p* = 0.220, *η*_P_^2^ = 0.21), and retinol (*p* = 0.121, *η*_P_^2^ = 0.31) were all similar to PLC.

## 4. Discussion

Cystic fibrosis is associated with a variety of systemic complications including vascular endothelial dysfunction [8, 9]. However, the mechanism(s) contributing to this dysfunction in CF have yet to be elucidated. To the best of our knowledge, this is the first study to investigate oxidative stress as a potential mechanism that contributes to vascular endothelial dysfunction in CF. Findings from the present study support our hypothesis that a single dose of an AOC elicits a significant improvement in vascular endothelial function compared to no change with placebo (Figure 2). In addition, AOC treatment significantly prevented the reduction in circulating concentrations of *α*-tocopherol and tended to decrease LOOH compared to the placebo (Figure 3). Together, these findings provide strong mechanistic evidence that oxidative stress contributes to vascular dysfunction in patients with CF.

Recently, our group provided the first evidence of both conduit- and microvascular endothelial dysfunction in young patients with CF [8, 9]. The FMD test not only is reproducible in patients with CF [33] but also allows for noninvasive assessment of vascular endothelial function [4–6] and, importantly, NO bioavailability [7]. NO-dependent vasodilation is perhaps the most important signaling function of the endothelium due to the protective effect against the development of atherosclerosis [34]. Oxidative stress, an established characteristic of CF [10–16], can negatively impact endothelial function as NO rapidly reacts with superoxide [35, 36] and reduces NO bioavailability [17].

The etiology of oxidative stress in CF is related to both pulmonary and nonpulmonary manifestations of the disease. First, CF directly causes chronic pulmonary infection that not only contributes to a persistently elevated proinflammatory immune response but also results in overproduction of reactive oxygen species (ROS) by activated leukocytes [16, 18]. Although basal inflammation (i.e., CRP) was higher in patients compared to controls (Table 1), inflammatory biomarkers were not assessed as we did not anticipate any changes in systemic inflammation following a single AOC. Second, the dysfunctional cystic fibrosis transmembrane regulator (CFTR) gene contributes to pancreatic insufficiency and nutrient malabsorption [37] which leads to diminished secretion of pancreatic enzymes, dysfunctional lipid digestion, and ultimately, reduced absorption of fat-soluble vitamins. Indeed, several of these essential vitamins serve as antioxidants (e.g., vitamins A and E), and their impaired absorption likely contributes to oxidative stress. For this reason, many patients with CF are prescribed with daily fat-soluble vitamins (e.g., AquADEK); however, the AOC used in the present study may have even greater therapeutic potential for several reasons. First, *α*-tocopherol, the main lipid chain-breaking antioxidant, is maintained only in the presence of ascorbic acid [38] and rapid reactions between the two encourage recycling of *α*-tocopherol. Further, *α*-lipoic acid, a powerful dual-phase (aqueous and lipid) antioxidant [39], aids in the reduction of dehydroascorbic acid to ascorbic acid [40], highlighting its recycling ability and involvement in complex antioxidant networks. Thus, the combination of antioxidants used in our AOC works synergistically to combat oxidative stress. Indeed, utilizing a placebo-controlled within-patient experimental design, our data indicate that the AOC significantly prevented the reduction in *α*-tocopherol and tended to reduce indices of oxidative stress (Figure 3). In addition, these data support the ability of the CF gut to absorb the AOC into circulation. Encouragingly, FMD following the AOC in both children and adults was restored to a value similar to that of healthy controls, possibly due to improved ROS buffering capacity and an increase in NO bioavailability (Table 2). Although outside the scope of the present investigation and unlikely to impact the findings following a single experimental treatment, we cannot rule out the potential effects of CFTR genotype and modulator therapies acting on the vasculature. The CFTR gene is expressed on endothelial cells and may impact vascular reactivity independent of oxidative stress balance. Further studies are certainly warranted to clarify the potential influence of CFTR therapies on vascular endothelial function in CF.

Taken together, these observations provide compelling evidence to support the role of oxidative stress as a key contributor to vascular endothelial dysfunction in CF. Our findings suggest that an oral AOC is capable of reducing oxidative stress and may provide therapeutic benefit for patients with CF. Indeed, the present findings warrant further investigation to determine the impact of extended (i.e., >6 months) antioxidant treatment on oxidative stress and vascular function in this patient population.

### 4.1. Clinical Significance

The development of cardiovascular disease (CVD) is closely tied to endothelial dysfunction [41], and a 1% decrease in FMD is associated with an ~8% increase in the risk of future cardiovascular events [42]. In the present study, the 1.9% increase in FMD observed following the AOC treatment translates to a ~15% risk reduction for future cardiovascular events. Beyond the potential impact antioxidant treatments may have on CVD risk reduction in CF, previous work from our group has implicated endothelial dysfunction as a contributor to exercise capacity [8] and exercise blood flow regulation [43], key areas of concern for patients with CF given that exercise intolerance is an independent predictor of mortality in this population [44, 45]. Thus, the therapeutic potential of antioxidant treatments in CF to improve endothelial function may have far-reaching clinical implications and warrants further investigation.

## 5. Conclusions

This is the first known study to investigate oxidative stress as a potential mechanism that contributes to vascular endothelial dysfunction in patients with CF. Importantly, ingestion of a single oral antioxidant cocktail treatment in patients not only improved FMD but also restored endothelial function to the value of healthy controls. Collectively, the improvement in oxidative stress balance coupled with the improved FMD following the antioxidant cocktail treatment indicates that oxidative stress is an important contributor to endothelial dysfunction in CF. Future studies are needed to determine if chronic antioxidant administration can lead to sustained improvements in endothelial function in patients with CF.

## Figures and Tables

**Table 1:** Participant characteristics, clinical laboratory markers, and pulmonary function in patients with CF and controls.

Variable	CF	Controls	*p* value
*N*	18	18	
Sex (M/F)	8/10	8/10	
Age (y)	18.8 ± 9.4	15.7 ± 5.2	0.227
Height (cm)	158 ± 15	163 ± 15	0.337
Weight (kg)	53.4 ± 15.9	52.5 ± 16.5	0.868
BMI (kg/m^2^)	20.8 ± 3.3	19.3 ± 3.9	0.225
Body fat (%)	22.0 ± 6.3	22.3 ± 8.2	0.902
SBP (mmHg)	108 ± 12	108 ± 16	0.915
DBP (mmHg)	60 ± 7	63 ± 8	0.133
Resting SpO_2_ (%)	97.9 ± 1.5	99.0 ± 0.6	**0.005**
Clinical laboratory markers			
TC (mg/dL)	127 ± 22	148 ± 26	**0.009**
HDL (mg/dL)	42 ± 12	55 ± 11	**0.002**
LDL (mg/dL)	66 ± 16	73 ± 36	0.508
Triglycerides (mg/dL)	88 ± 31	73 ± 27	0.144
Glucose (mg/dL)	86 ± 14	84 ± 9	0.658
TC : HDL	3.2 ± 0.7	2.8 ± 0.6	0.075
hsCRP	2.31 ± 2.33	0.51 ± 0.33	**0.003**
Pulmonary function			
FVC (L)	3.66 ± 1.25	4.10 ± 1.28	0.291
FEV_1_ (L)	2.78 ± 1.00	3.53 ± 0.98	**0.025**
FEV_1_ (% predicted)	88.0 ± 18.1	104.4 ± 9.6	**0.002**
FEV_1_/FVC (%)	75.5 ± 9.2	87.6 ± 7.3	**<0.001**
FEF_25-75_ (L/s)	2.47 ± 1.23	4.06 ± 1.27	**<0.001**

Values are the mean ± SD. BMI = body mass index; SBP = systolic blood pressure; DBP = diastolic blood pressure; SpO_2_ = oxygen saturation; TC = total cholesterol; HDL = high-density lipoprotein; LDL = low-density lipoprotein; hsCRP = high-sensitivity C-reactive protein; FVC = forced vital capacity; FEV_1_ = forced expiratory volume in 1 second; FEF_25-75_ = forced expiratory flow.

**Table 2:** Parameters of the FMD test in patients with CF completing the AOC treatment (CF-AOC; *n* = 18), placebo condition (CF-PLC; *n* = 9), or healthy controls (*n* = 18).

Variable	CF-AOC		CF-PLC		Controls
	Pre	Post	Pre	Post	
Baseline diameter (cm)	0.306 ± 0.055	0.302 ± 0.053	0.328 ± 0.049	0.325 ± 0.051	0.294 ± 0.043
Peak diameter (cm)	0.323 ± 0.056	0.324 ± 0.054	0.348 ± 0.048	0.344 ± 0.053	0.315 ± 0.052
FMD absolute change (cm)	0.017 ± 0.008	0.022 ± 0.011^∗^	0.021 ± 0.012	0.018 ± 0.008	0.022 ± 0.012
Shear rate (s^−1^, AUC)	58,273 ± 29,735	51,089 ± 24,393	52,527 ± 25,003	43,242 ± 30,749	45,737 ± 10,735
FMD/shear (%/s^−1^, AUC)	0.11 ± 0.06	0.15 ± 0.06	0.14 ± 0.09	0.16 ± 0.08	0.16 ± 0.06^†^
Time to peak (s)	58.1 ± 28.1	45.3 ± 19.9	70.8 ± 35.3	41.4 ± 16.4	38.1 ± 10.1^†‡^

Values are the mean ± SD. FMD = flow-mediated dilation. ^∗^Significant pre- to posttreatment change versus CF-PLC (*p* < 0.05); ^†^significant difference versus Pre in CF-AOC (*p* < 0.05); ^‡^significant difference versus Pre in CF-PLC (*p* = 0.024).

**Table 3:** Biomarkers of oxidative stress and lipid soluble antioxidants in patients with CF following the AOC treatment and placebo (PLC) condition (*n* = 9).

Variable	CF-AOC			CF-PLC		
	Pre	Post	Change	Pre	Post	Change
8-Isoprostane (pg/mL)	9.9 ± 3.8	11.4 ± 4.5	1.5 ± 1.6	10.9 ± 6.8	11.6 ± 7.5	0.8 ± 1.5
Nitrotyrosine (nM)	172.7 ± 69.4	180.1 ± 72.3	7.4 ± 25.3	200.1 ± 84.8	215.5 ± 52.2	15.4 ± 84.6
*γ*-Tocopherol (*μ*M)	2.16 ± 1.16	1.97 ± 0.78	−0.19 ± 1.34	2.12 ± 0.95	1.58 ± 0.81	−0.54 ± 0.84
Retinol (*μ*M)	2.36 ± 0.83	2.20 ± 0.72	−0.16 ± 0.93	2.22 ± 0.99	2.10 ± 0.94	−0.12 ± 1.11

Values are the mean ± SD.

## Data Availability

The data used to support the findings of this study are available from the corresponding author upon request.
